# Microstructural Evolution and Enhanced Macroscopic Properties of La-Doped TiO_2_-SiO_2_ Composite Films Under Gradient Annealing

**DOI:** 10.3390/mi17050617

**Published:** 2026-05-17

**Authors:** Yanbo Yuan, Li Zhang, Lei Li, Mengyang Wang, Wenjun Wang, Lin Wang

**Affiliations:** College of Mechanics and Aeronautics, Inner Mongolia University of Technology, Hohhot 010051, China; yyb2624095231@gmail.com (Y.Y.); leillt@163.com (L.L.); 15774737125@163.com (M.W.); w2087853475@163.com (W.W.); 18347980766@163.com (L.W.)

**Keywords:** La-doped TiO_2_-SiO_2_ composite films, magnetron sputtering, annealing, self-cleaning potential, stability

## Abstract

In this study, La-doped TiO_2_-SiO_2_ composite films were deposited on glass substrates by radio-frequency magnetron sputtering. The evolution of microstructure and macroscopic properties was systematically investigated across an annealing temperature range of 350–650 °C. The results show that the La-doped TiO_2_-SiO_2_ composite structure effectively suppresses abnormal grain growth and delays the anatase-to-rutile phase transition, thereby improving the films’ high-temperature structural stability. Notably, the composite film annealed at 550 °C (LS-550) exhibits the highest anatase crystallinity and forms a dense, smooth (RMS = 1.37 nm), crack-free nanocrystalline network. In terms of wettability, the improved hydrophilicity is attributed to the combined effects of La incorporation and hydrophilic silanol (Si-OH) groups in the amorphous SiO_2_ phase. As a result, the water contact angle of the LS-550 film decreases dramatically to 28.0°, indicating excellent hydrophilicity. Moreover, the LS-550 film demonstrates an optimal photocatalytic degradation efficiency of approximately 76% for methylene blue, significantly outperforming the pure TiO_2_ film. Furthermore, the enhanced mechanical performance is associated with the combined effects of the SiO_2_-containing amorphous phase and the finer microstructure induced by La incorporation. Consequently, the critical load (Lc) of the LS-550 film reaches 75.64 mN, significantly exceeding that of the pure TiO_2_ film annealed at the same temperature (61.25 mN). In summary, the composite film annealed at 550 °C concurrently achieves high crystallographic thermal stability, robust interfacial mechanical durability, excellent surface hydrophilicity, and enhanced photocatalytic activity, thereby offering practical guidance for developing TiO_2_-based coatings with self-cleaning potential for high-rise building curtain walls.

## 1. Introduction

The manual cleaning of exterior windows and curtain-wall glass in high-rise buildings entails prohibitive costs and substantial safety risks [1]. Endowing glass surfaces with self-cleaning functionality can significantly reduce the need for manual maintenance while effectively preserving their aesthetic appearance and optical transmittance [2]. In the photovoltaic sector, dust accumulation likewise severely compromises the power-generation efficiency of solar modules [3]. By reducing surface soiling and facilitating water-spreading removal of contaminants, self-cleaning coatings can help maintain the optical performance of photovoltaic cover glass [4]. Among various self-cleaning materials, titanium dioxide (TiO_2_) is the most widely used [5]. Its self-cleaning behavior mainly arises from two synergistic effects: first, TiO_2_ photocatalytically oxidizes and decomposes organic contaminants under ultraviolet (UV) irradiation [6]; second, its photoinduced superhydrophilicity allows rainwater to spread across the surface as a continuous film, thereby removing inorganic particulate matter [7]. Therefore, the development of highly efficient and durable TiO_2_ self-cleaning films is of great practical importance for engineering applications in building facades and photovoltaic glass [8].

From a microstructural perspective, the crystalline phase and defect structure of TiO_2_ largely determine its physicochemical behavior [9]. Although the anatase phase generally exhibits superior photocatalytic activity and hydrophilicity compared to rutile [10], it is highly susceptible to an irreversible transformation into rutile during high-temperature thermal treatment, inevitably leading to a deterioration in self-cleaning performance [11]. Previous studies have demonstrated that the phase-transformation kinetics of TiO_2_ films are strongly governed by factors such as grain size, lattice defects, internal stress, and elemental modification [12].

Magnetron sputtering has become an attractive route for TiO_2_ thin-film deposition due to its capability for large-area uniform coating and precise control over processing parameters [13]. Alongside sputtering-based techniques, innovative low-temperature approaches have also been developed for fabricating compact TiO_2_ layers. For example, UV-assisted TiBr_4_ chemical bath deposition has proven effective in yielding dense TiO_2_ films under relatively mild conditions, offering an alternative pathway for compact layer preparation [14]. In the present study, however, magnetron sputtering was specifically adopted as it facilitates the simultaneous co-introduction of La and SiO_2_ and allows for a systematic examination of the annealing-dependent structural evolution. By carefully tailoring the deposition parameters and annealing temperatures, the crystallographic composition and microstructural features of the TiO_2_ composite films can be effectively modulated [15].

In recent years, extensive efforts have been devoted to modifying TiO_2_ thin films through deposition-parameter optimization and elemental doping to improve their structural and photocatalytic properties [16,17]. Anita et al. reported that SiO_2_ doping can significantly retard the anatase-to-rutile phase transition in TiO_2_ powders by introducing lattice strain and reducing grain size [18]. Moreover, Mishra and Kannan experimentally demonstrated in a TiO_2_-SiO_2_ binary oxide system that the incorporation of SiO_2_ suppresses grain growth and constrains lattice coarsening, thereby delaying the phase transformation [19]. In the context of rare-earth doping, the incorporation of La^3+^ into the TiO_2_ lattice not only regulates grain size but also exerts a pronounced positive effect on its optoelectronic properties [20,21]. Based on first-principles calculations, previous studies have suggested that La doping can enhance the structural stability and photocatalytic activity of TiO_2_ by modifying the local lattice environment and promoting defect formation [22]. Likewise, experimental studies by Verma and Singh showed that La doping refines the crystallite size of TiO_2_, extends its optical absorption into the visible-light region, and markedly improves its photocatalytic degradation efficiency, resulting in a higher degradation rate for organic dyes than for undoped counterparts [23].

Nevertheless, although single-element doping strategies can improve specific properties, they generally fail to simultaneously optimize the high-temperature crystallographic stability, surface wettability, and mechanical durability of TiO_2_ films. In this context, a promising strategy is to combine the spatial confinement effect associated with the amorphous SiO_2_ phase with the structural modification induced by La incorporation. Through the synergistic interaction of these two effects, high-temperature grain coarsening can be effectively suppressed. At the same time, the interfacial adhesion and surface wettability of the films can be improved. However, systematic studies on the microstructural evolution of La-doped TiO_2_-SiO_2_ composite films under gradient annealing temperatures remain scarce, and the synergistic effects responsible for their enhanced hydrophilicity and film–substrate adhesion have yet to be fully clarified.

To address this gap, La-doped TiO_2_-SiO_2_ composite films were deposited on glass substrates by radio-frequency magnetron sputtering. To systematically investigate the thermally driven evolution of structure and properties, the as-deposited films were annealed at 350, 450, 550, and 650 °C, with a pure TiO_2_ film annealed at 550 °C used as a reference. A series of structural, optical, and mechanical analyses, including X-ray diffraction (XRD), scanning electron microscopy (SEM), energy-dispersive X-ray spectroscopy (EDS), atomic force microscopy (AFM), water contact angle measurements, and nano-scratch testing, were performed to investigate the effects of annealing temperature on the crystallographic phase evolution, surface morphology, elemental distribution, wettability, and interfacial mechanical properties of the films. Consequently, this work focuses on evaluating the combined structural and mechanical benefits of the La-doped TiO_2_-SiO_2_ composite structure on the microstructure and macroscopic performance of TiO_2_ films, thereby providing an experimental basis for the design of TiO_2_-based coatings with self-cleaning potential for high-rise building facades and photovoltaic glass encapsulation.

## 2. Experimental Section

### 2.1. Film Preparation

In this study, the composite films were deposited using a JGP-450 high-vacuum multifunctional magnetron sputtering system (Shenyang Scientific Instrument Co., Ltd., Chinese Academy of Sciences, Shenyang, China). K9 optical glass substrates with dimensions of 20 mm × 20 mm × 2 mm were used [24]. Before deposition, the substrates were ultrasonically cleaned successively in acetone and anhydrous ethanol for 15 min each, thoroughly rinsed with deionized water, dried under a stream of high-purity nitrogen, and immediately transferred to the vacuum chamber [25].

A dual-target co-sputtering strategy was employed to achieve La-doped TiO_2_-SiO_2_ composite films [26,27]. The SiO_2_-TiO_2_ composite ceramic target (60 mm in diameter and 3 mm in thickness) was fabricated by sintering high-purity TiO_2_ (99.99%) and SiO_2_ (99.99%) powders, with the SiO_2_ doping concentration precisely controlled in the range of 8–10% [28]. A high-purity metallic La target (99.95%) was used as the lanthanum source. The target-to-substrate distance was fixed at 80 mm for both targets [29].

**Figure 1:**
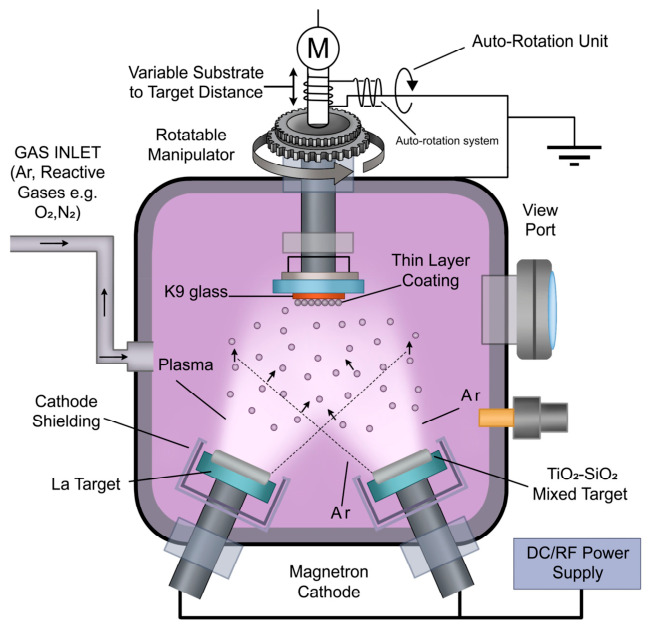
Schematic diagram of dual-target magnetron sputtering coating.

**Figure 2:**
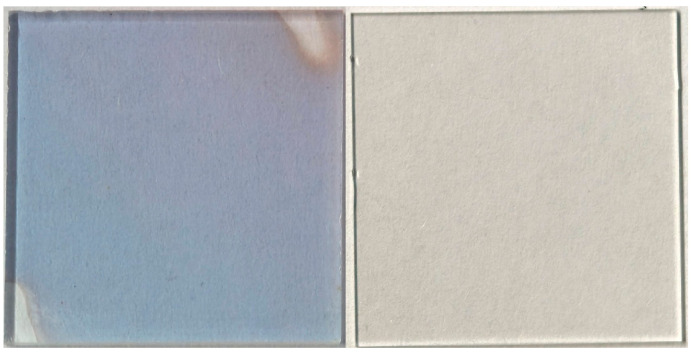
Pure titanium dioxide film (**left**) and lanthanum-doped titanium dioxide–silicon dioxide composite film (**right**).

Before gas introduction, the sputtering chamber was evacuated to a base pressure of 5.0 × 10^−5^ Pa. High-purity argon (99.999%) was then introduced at a flow rate of 35 sccm, and the working pressure was maintained at 1.0 Pa [30]. To remove surface contaminants and ensure target stability, both the SiO_2_-TiO_2_ composite target and the metallic La target were pre-sputtered for 15 min under identical conditions before deposition [31]. During film growth, radio-frequency (RF) power was applied exclusively to the SiO_2_-TiO_2_ composite target at 120 W, whereas direct-current (DC) power was supplied solely to the La target at 25 W, thereby enabling independent control of dopant incorporation. The substrates were maintained at room temperature and rotated at 20 rpm throughout the 2 h deposition process to ensure uniform film thickness and composition [32]. A schematic illustration of the deposition process is shown in Figure 1. The as-prepared films are shown in Figure 2.

### 2.2. Post-Deposition Annealing

The as-deposited films were thermally annealed in ambient air using an OTF-1200X tube furnace (Hefei Kejing Materials Technology Co., Ltd., Hefei, China). The annealing treatment was carried out at gradient temperatures of 350, 450, 550, and 650 °C for 2 h at a constant heating rate of 5 °C/min, followed by natural cooling to room temperature in the furnace [33]. For comparison, a pure TiO_2_ film was also prepared and annealed at 550 °C under the same conditions. The detailed nomenclature of the resulting samples is listed in Table 1.

This temperature gradient was selected based on the crystallization behavior and phase-transition characteristics of TiO_2_ during annealing. In general, 350 °C corresponds to the initial stage of the amorphous-to-anatase transformation, while 450–550 °C is considered the main crystallization range for anatase. Among these temperatures, 550 °C is commonly regarded as a critical point at which pure TiO_2_ begins to undergo the irreversible anatase-to-rutile transition [28]. Therefore, this annealing schedule was adopted to examine how the La-doped TiO_2_-SiO_2_ composite structure influences the formation and stability of the anatase phase and suppresses its transformation to rutile [34,35]. For comparison, a pure TiO_2_ film annealed at 550 °C was chosen as the reference sample, because this temperature is the most representative point for revealing the difference between pure TiO_2_ and the composite films. At this stage, the phase stability, grain-growth behavior, and related performance changes of pure TiO_2_ become more pronounced, making it suitable for evaluating the synergistic effects of La doping and SiO_2_ incorporation.

### 2.3. Characterization and Interfacial Mechanics Evaluation

The crystallographic structures of the films were characterized using an X-ray diffractometer (XRD, D/max-2500/PC, Rigaku Corporation, Tokyo, Japan) with Cu Kα radiation (λ = 0.15406 nm), operated at 40 kV and 100 mA. To enhance the diffraction signal from the films while reducing the amorphous background contribution from the glass substrate, grazing-incidence X-ray diffraction (GIXRD) was employed [36]. The measurements were performed with a fixed incidence angle of 1.5°, over a 2θ range of 5–90° at a step size of 0.02°. For quantitative analysis, the diffraction pattern of the blank K9 glass substrate was measured separately under the same GIXRD conditions and subtracted from each sample pattern. Based on the corrected patterns, the total integrated area of each sample was first determined, and the integrated areas of the anatase and rutile diffraction peaks were then calculated separately. The anatase and rutile crystallinity indices were defined as the ratios of their respective integrated peak areas to the total integrated area of the corrected pattern.

The surface morphologies of the films were examined using a field-emission scanning electron microscope (SEM, SU8010, Hitachi, Japan) operated at an accelerating voltage of 5 kV and an emission current of 10 μA. Before observation, the sample surfaces were sputter-coated with a thin layer of gold to improve electrical conductivity. An energy-dispersive X-ray spectroscopy (EDS, Oxford X-Max^50^, Oxford Instruments, Abingdon, High Wycombe, UK) detector integrated with the SEM was used to analyze the elemental composition and distribution at an accelerating voltage of 15 kV with an acquisition time of 120 s. Quantitative elemental analysis was performed using the ZAF correction method to determine elemental composition by weight percentage (wt.%). To further evaluate the spatial uniformity of elemental distribution on the film surface, including La, Si, Ti, and O, elemental mapping was carried out over an area of 10 μm × 10 μm at a resolution of 512 × 400 pixels [37].

The three-dimensional surface topography and nanoscale roughness of the films were characterized using an atomic force microscope (AFM, Multimode 8, Bruker, Berlin, Germany) operated in tapping mode. An RTESP silicon probe with a resonance frequency of approximately 300 kHz and a spring constant of 40 N/m was used for scanning. Measurements were performed over scan areas of 10 μm × 10 μm at a scan rate of 1.0 Hz and a resolution of 512 × 512 pixels [38]. The root-mean-square roughness (Rq) and arithmetic average roughness (Ra) were subsequently calculated using NanoScope Analysis 3.0 software.

To quantitatively evaluate the hydrophilicity of the films, the static water contact angles (WCA) on the film surfaces were measured using a JC2000D2 contact angle goniometer (Shanghai, China). All measurements were conducted at an ambient temperature of 25 °C and a relative humidity of 45%.

The photocatalytic activity of the La-doped TiO_2_-SiO_2_ composite films was evaluated via methylene blue (MB) degradation. Films were immersed in 30 mL of MB solution (10 mg·L^−1^) and kept in the dark for 30 min to establish adsorption–desorption equilibrium. Irradiation was provided by a 300 W xenon lamp (λ > 300 nm, 100 mW·cm^−2^). Aliquots were sampled at regular intervals up to 60 min, and temporal MB concentrations were determined via UV-Vis absorbance at 665 nm. Based on the initial concentration of the MB solution (C_0_) and the concentration after an irradiation time t (C_t_), the degradation rate was calculated as follows.

$$Degradation\;rate\left(\%\right)=\frac{C_{0}-C_{t}}{C_{0}}\times 100\%\;$$

The interfacial adhesion strength between the composite films and the glass substrates was systematically evaluated using a Nano Indenter G200 (Agilent, Santa Clara, CA, USA). The instrument was equipped with a diamond conical indenter with a tip radius of 5 μm and a cone angle of 90°. The scratch-test parameters were set as follows: a scratch length of 500 μm, a normal load linearly increased from 0 to 100 mN, and a constant scratch velocity of 10 μm/s. To ensure precise synchronization between the load increase and the scratch displacement, the linear loading rate was maintained at 2 mN/s [39].

## 3. Results and Discussion

### 3.1. X-Ray Diffraction (XRD) Analysis

**Figure 3:**
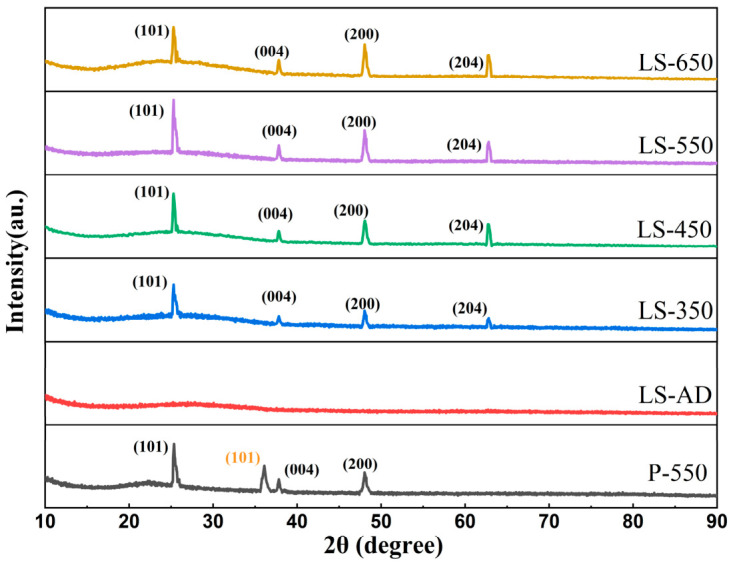
XRD patterns of La-doped TiO_2_-SiO_2_ composite films at different annealing temperatures.

Figure 3 shows the XRD patterns of the La-doped TiO_2_-SiO_2_ composite films annealed at different temperatures. The as-deposited composite film (LS-AD) exhibits a broad amorphous halo, in which only the diffuse scattering peak from the glass substrate can be discerned, indicating the absence of a long-range ordered crystalline structure in the unannealed film [40]. After annealing at 350 °C (LS-350), weak diffraction peaks appear at 2θ ≈ 25.3°, 37.8°, 48.0°, and 62.7°, which can be indexed to the anatase (101), (004), (200), and (204) planes, respectively, marking the onset of crystallization [41]. As the annealing temperature increases to 450 °C (LS-450) and 550 °C (LS-550), these diffraction peaks become progressively sharper and more intense. The anatase (101) peak reaches its highest intensity in LS-550, indicating that anatase development is most pronounced at this temperature [21]. The quantitative XRD results listed in Table 2 show the same trend. The anatase crystallinity index increases from 28.0% for LS-350 to 45.9% for LS-450 and reaches a maximum of 49.7% for LS-550, followed by a decrease to 41.6% for LS-650. This trend is consistent with the evolution of the diffraction peaks and indicates that annealing up to 550 °C promotes the formation and ordering of the anatase phase in the composite films, whereas a further increase to 650 °C slightly weakens this effect. The slightly lower peak intensity of LS-650 compared with LS-550 may be related to dopant redistribution at grain boundaries and the associated local lattice distortion under excessive thermal input [42]. Nevertheless, no characteristic rutile peak is observed in LS-650, indicating that the composite system still maintains good phase stability at elevated temperatures.

The pure TiO_2_ control sample annealed at 550 °C (P-550) already shows a distinct rutile (101) diffraction peak at 2θ ≈ 36.1°. Its anatase and rutile crystallinity indices are 32.3% and 12.4%, respectively. In contrast, LS-550 exhibits a markedly higher anatase crystallinity index and no detectable rutile diffraction. Combined with the temperature-dependent evolution observed in the composite series, these results indicate that the La-doped TiO_2_-SiO_2_ composite structure delays the phase transformation of TiO_2_. The amorphous SiO_2_ phase likely limits direct intergranular contact and suppresses excessive grain coarsening during annealing [43], while La incorporation introduces local lattice distortion or related defect states that hinder atomic migration and slow the structural evolution associated with the anatase-to-rutile transformation [33,44]. Together, these effects account for the improved crystallographic stability and the better retention of the anatase phase in the composite films.

### 3.2. Surface Morphology (SEM) and Elemental Analysis (EDS/Mapping)

#### 3.2.1. Surface Morphology (SEM) Analysis

**Figure 4:**
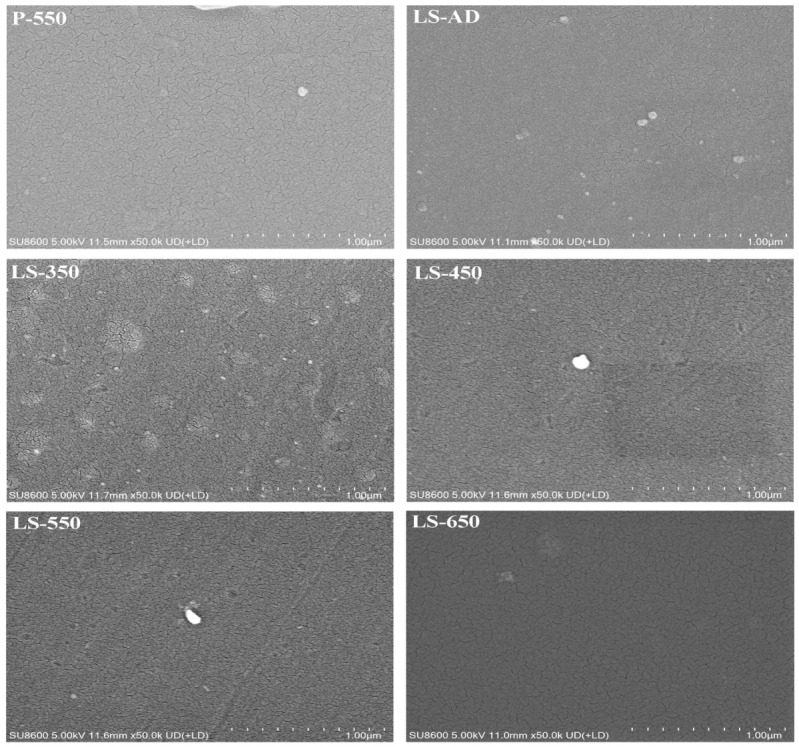
SEM images of the films at different annealing temperatures.

Figure 4 presents the SEM micrographs of the La-doped TiO_2_-SiO_2_ composite films subjected to gradient annealing temperatures. These images illustrate the morphological evolution of the films under the combined influence of the composite structure and thermal treatment. The as-deposited film (LS-AD) exhibits a featureless surface, which is consistent with its amorphous nature. As the annealing temperature increases to 350 °C and 450 °C, uniformly distributed nanocrystallites gradually appear on the film surface. At 550 °C (LS-550), the film develops a dense, homogeneous, and crack-free nanocrystalline network, consistent with the enhanced crystallinity indicated by the XRD results. However, upon further annealing at 650 °C (LS-650), slight localized grain coalescence and coarsening are observed, which can be attributed to thermally induced structural instability due to excessive heat input [42].

In sharp contrast, the pure TiO_2_ control sample annealed at 550 °C (P-550) exhibits severe grain coarsening accompanied by an extensive network of reticulated microcracks. This pronounced morphological difference indicates that the La-doped TiO_2_-SiO_2_ composite structure effectively suppresses excessive grain growth and preserves film integrity during annealing. At the microscopic level, this behavior can be associated with the combined effects of La incorporation and the SiO_2_-containing amorphous phase on grain-boundary migration [44]. At the macroscopic level, the finer, more compact microstructure of the composite films helps alleviate stress concentrations generated during high-temperature annealing [12], thereby reducing the tendency for microcrack formation and improving the films’ compactness and structural integrity.

#### 3.2.2. Elemental Composition and Distribution (EDS/Mapping) Analysis

**Figure 5:**
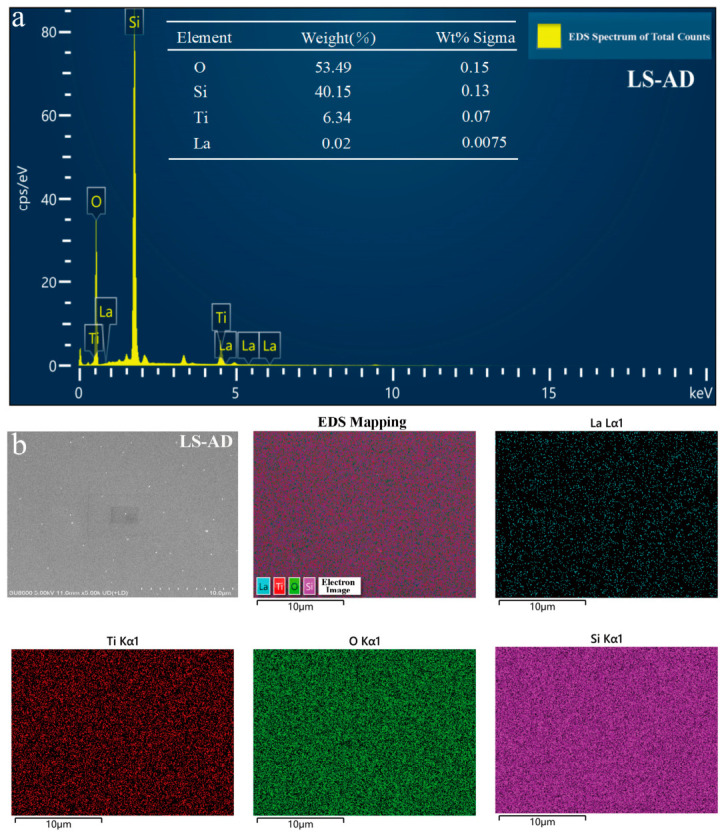
EDS spectra and elemental mapping of the as-deposited composite film (LS-AD). (**a**) EDS spectrum of the as-deposited composite film (LS-AD). (**b**) Elemental mapping of the as-deposited composite film (LS-AD).

Figure 5 presents the energy-dispersive X-ray spectroscopy (EDS) spectra and corresponding elemental mapping results of the as-deposited composite film (LS-AD). The EDS spectra exhibit characteristic signals from Ti, O, Si, and La, with no obvious impurity peaks. Semi-quantitative analysis indicates that the mass fraction of La is approximately 0.02%, corresponding to 0.315% relative to the Ti content [21]. These results confirm the successful incorporation of the rare-earth element La into the film matrix. The prominent Si signal, with a relatively high mass fraction of 40.15%, is mainly attributed to the combined contribution of the incorporated SiO_2_ phase and the underlying glass substrate. Given the strong background signal from the substrate, this semi-quantitative approach accurately reflects the relative elemental presence rather than absolute bulk concentrations. Furthermore, the elemental mapping images show that Ti, O, Si, and trace amounts of La are distributed relatively uniformly across the scanned area, with no evident local segregation or elemental agglomeration. Collectively, these findings demonstrate that the employed magnetron sputtering process enables the uniform co-deposition of multiple elements, thereby ensuring good chemical homogeneity throughout the composite film.

### 3.3. Atomic Force Microscopy (AFM) Analysis

**Figure 6:**
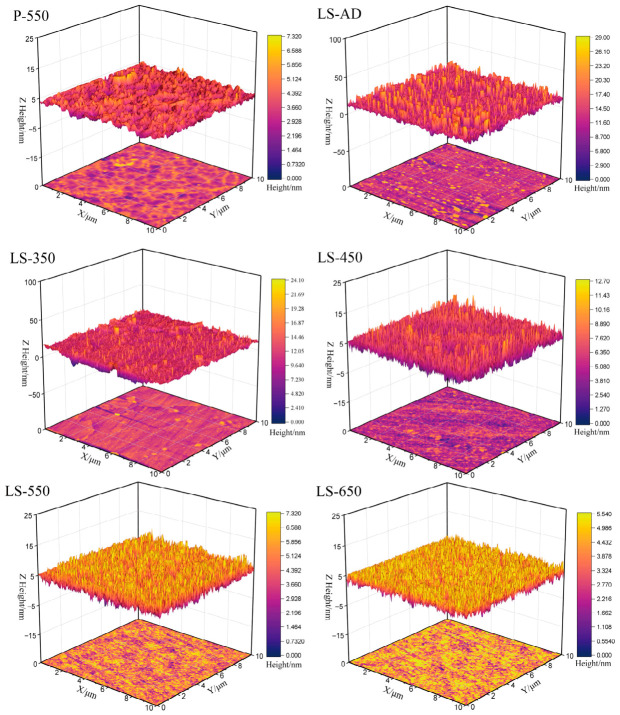
AFM images of the film at different annealing temperatures.

Figure 6 presents the three-dimensional surface topographies of the films annealed at different temperatures, and the corresponding surface roughness data are summarized in Table 3. AFM measurements were performed to quantitatively evaluate the surface nanoroughness. The as-deposited film (LS-AD) exhibits the most pronounced surface undulations, with a root-mean-square (RMS) roughness of 4.60 nm and an arithmetic average roughness (Ra) of 3.28 nm. After thermal treatment, the surface roughness decreases markedly. Upon annealing at 350 °C (LS-350), the RMS roughness drops to 1.92 nm. As the annealing temperature further increases to 450–650 °C, the roughness remains relatively low, with RMS values of 1.25 nm, 1.37 nm, and 1.22 nm for LS-450, LS-550, and LS-650, respectively. This overall trend is consistent with the XRD results, indicating that annealing promotes structural rearrangement and facilitates the gradual transformation of the amorphous film into a denser anatase-dominant structure. Such evolution helps reduce surface irregularities and microdefects [45], thereby leading to a smoother and more compact surface morphology.

To further clarify the effect of the composite structure on surface morphology, a comparative analysis was performed between the composite and pure TiO_2_ films annealed at 550 °C. The results show that the pure TiO_2_ film (P-550) exhibits higher surface roughness, with an RMS value of 1.70 nm and an Ra value of 1.20 nm. In contrast, the composite film (LS-550) displays a smoother surface, with corresponding RMS and Ra values of 1.37 nm and 1.09 nm, respectively. This difference indicates that the La-doped TiO_2_-SiO_2_ composite structure effectively suppresses excessive surface coarsening during annealing. At elevated temperatures, grain-boundary migration in pure TiO_2_ proceeds more readily, making the grains more susceptible to abnormal growth and agglomeration-induced coarsening [46], which consequently increases the surface roughness of P-550. In contrast, in the composite system, the SiO_2_-containing amorphous phase and La incorporation help restrain grain growth [43]. As a result, the LS-550 film retains a relatively dense nanoscale surface structure while exhibiting enhanced crystallinity [42].

### 3.4. Contact Angle and Wettability Analysis

**Figure 7:**
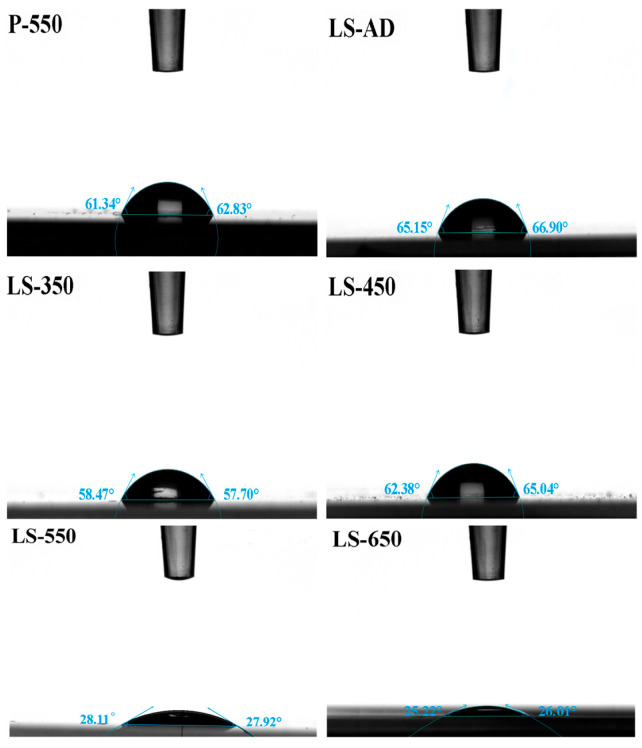
Contact angle profiles of films annealed at different temperatures.

Figure 7 presents the static water contact angle (WCA) values of the films under different annealing temperatures. The WCA measurements clearly reveal the evolution of the films’ wettability. The as-deposited film (LS-AD) exhibits an initial WCA of approximately 66.0°. After annealing at 350 °C (LS-350), the formation of the anatase phase reduces the WCA to 58.1° [30]. Notably, at 450 °C (LS-450), the WCA increases again to 63.7°. In conjunction with the AFM results and the classical Wenzel wetting model [47], this phenomenon can be attributed to the pronounced reduction in surface nanoroughness during the early stage of crystallization, which weakens the capillary amplification effect of the surface microstructure [48]. However, as the annealing temperature further increases to 550 °C (LS-550) and 650 °C (LS-650), the WCA decreases sharply to 28.0° and 25.6°, respectively, indicating excellent hydrophilicity [49]. In contrast, the pure TiO_2_ control film annealed at 550 °C (P-550) maintains a relatively high WCA of 62.1°, indicating that the composite strategy effectively improves film wettability.

The excellent hydrophilicity of the composite films is associated with the combined effects of crystallographic evolution and surface chemical characteristics. First, the improved anatase crystallinity provides more hydrophilic and reactive surface sites [50]. Second, La incorporation modifies the local lattice environment and facilitates the formation of surface defect sites favorable for water adsorption [51]. In addition, the presence of the SiO_2_-containing amorphous phase introduces hydrophilic silanol groups (Si-OH) [52], thereby enhancing the affinity of the surface for water molecules. The combined effect of these factors accounts for the markedly improved wettability of the composite films compared with pure TiO_2_. The slight further decrease in WCA observed for the LS-650 sample can be attributed to thermally induced surface compositional redistribution under excessive heat input, which further influences the macroscopic wetting behavior.

### 3.5. Photocatalytic Activity Testing

To further evaluate the macroscopic properties of the La-doped TiO_2_-SiO_2_ composite films under different gradient annealing temperatures, their photocatalytic degradation efficiency was tested using MB as the target pollutant. A blank experiment utilizing a bare glass substrate (denoted as Blank) was also conducted under identical conditions for comparison.

**Figure 8:**
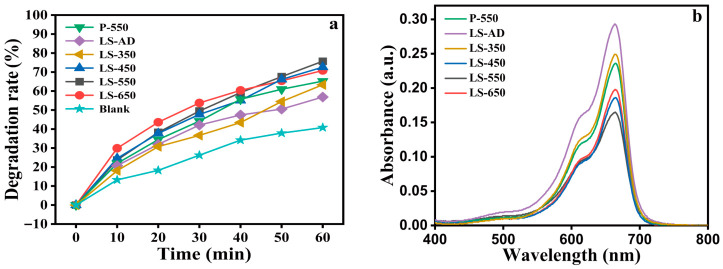
Photocatalytic performance of the La-doped TiO_2_-SiO_2_ composite films. Time-dependent degradation rates of methylene blue (MB) solution under full-spectrum irradiation for various annealed films and a blank glass substrate (**a**). UV-Vis absorbance spectra of the corresponding MB solutions after 60 min of reaction (**b**).

As illustrated in Figure 8a, the blank sample exhibits a moderate photolysis effect under irradiation, with the MB degradation rate reaching approximately 40% after 60 min. In contrast, all film-coated samples show significantly enhanced degradation rates, unequivocally confirming the intrinsic photocatalytic activity of the prepared composite films. The pristine film (P-550) and the as-deposited film (LS-AD) demonstrate degradation efficiencies of roughly 65% and 57%, respectively. Notably, with the introduction of gradient annealing, the photocatalytic activity initially improves and then slightly declines. The LS-550 sample exhibits the most superior degradation performance, reaching nearly 76% after 60 min, outperforming LS-450 (~72%) and LS-650 (~70%).

This trend is strongly corroborated by the UV-Vis absorbance spectra of the MB solutions after 1 h of reaction, as shown in Figure 8b. The characteristic absorption peak of MB at 665 nm displays the lowest intensity for the LS-550 sample, further validating its optimal photocatalytic capability. The significantly enhanced performance at 550 °C can be attributed to the favorable microstructural evolution induced by the specific annealing temperature, where the optimized crystallinity and phase composition likely provide more surface active sites and facilitate the efficient separation of photo-generated electron–hole pairs. Conversely, the slight reduction in photocatalytic efficiency observed at 650 °C may be linked to excessive crystallite growth. While higher annealing temperatures promote crystalline perfection, the excessively grown crystallites inevitably lead to a reduction in the specific surface area, thereby decreasing the density of available surface active sites for the photocatalytic reaction.

### 3.6. Mechanical Property Testing

**Figure 9:**
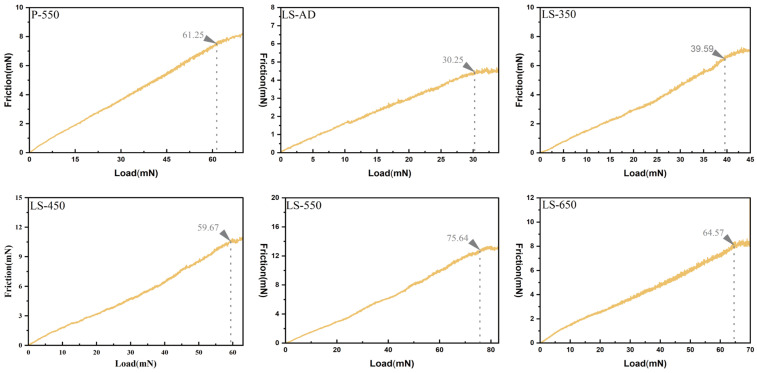
Adhesion strength of the film at different annealing temperatures.

Nano-scratch testing was employed to quantitatively evaluate the interfacial adhesion of the films, with the critical load (Lc) used as the evaluation criterion [53,54]. It should be noted that all composite films evaluated in this study were deposited under consistent sputtering parameters and a fixed deposition time of 2 h. Therefore, any deviations in the initial physical thickness among the as-deposited films are expected to be negligible. Consequently, the observed variations in interfacial adhesion across different samples can be largely attributed to the microstructural evolution induced by the subsequent gradient annealing. As shown in Figure 9, which summarizes the film–substrate adhesion behavior at different annealing temperatures, the as-deposited film (LS-AD) exhibits a relatively low Lc of 30.25 mN, reflecting weak interfacial bonding in the absence of post-deposition thermal treatment. With increasing annealing temperature, the Lc gradually increases, reaching 39.59 mN for LS-350, 59.67 mN for LS-450, and ultimately peaking at 75.64 mN for LS-550. This trend indicates that moderate annealing improves film–substrate adhesion, as thermally activated interfacial diffusion and structural densification help establish a more stable interfacial state. However, upon further annealing at 650 °C (LS-650), the Lc decreases to 64.57 mN. Compared with the pure TiO_2_ film annealed at the same temperature (P-550, Lc = 61.25 mN), the LS-550 sample exhibits markedly superior scratch resistance. This improvement in mechanical performance can be attributed to the combined effects of La and SiO_2_ on the films’ microstructure and interfacial response. Pure TiO_2_ is prone to residual internal stress accumulation due to a mismatch in the coefficient of thermal expansion (CTE) between the film and the glass substrate, which can readily induce brittle delamination under applied shear loading [55,56]. In contrast, the composite films exhibit a denser, finer-grained microstructure, which helps relieve local stress concentrations during cooling and scratch loading. At the same time, the SiO_2_-containing amorphous phase contributes to interfacial compliance [57], while the grain-refinement effect associated with La incorporation improves resistance to crack initiation and propagation [58]. The subsequent decline in the interfacial adhesion of the LS-650 sample can be attributed to microstructural deterioration under excessive thermal input, thereby weakening the beneficial effect observed at 550 °C.

## 4. Conclusions

In this study, we systematically investigated the microstructural evolution, macroscopic properties, and related synergistic effects of La-doped TiO_2_-SiO_2_ composite films deposited by magnetron sputtering and annealed at 350, 450, 550, and 650 °C. A series of characterizations, including XRD, SEM, EDS, AFM, water contact angle measurements, and nano-scratch testing, were carried out to examine the crystallographic evolution, surface morphology, wetting behavior, and interfacial adhesion of the films. The main findings are summarized as follows:

- **Microstructural and Morphological Stability:** Multiscale structural characterizations show that the La-doped TiO_2_-SiO_2_ composite structure suppresses abnormal grain growth and microcrack formation during high-temperature annealing. In addition, the composite films retain anatase-dominant characteristics over the investigated temperature range up to 650 °C, indicating improved resistance to the anatase-to-rutile transformation. Notably, the film annealed at 550 °C (LS-550) exhibits the most favorable overall balance between crystallization behavior and surface morphology, showing the highest anatase crystallinity, a dense, homogeneous, crack-free nanocrystalline network, and low surface roughness (RMS = 1.37 nm). These results demonstrate that the composite structure effectively improves the microstructural and morphological stability of TiO_2_ films during annealing.
- **Surface Wettability and Self-Cleaning Potential:** Water contact angle (WCA) measurements show that the composite strategy, combined with moderate-to-high-temperature annealing, promotes a clear transition toward a highly hydrophilic state, with the WCA decreasing to 28.0° or below for samples annealed at 550 °C and above. Furthermore, photocatalytic degradation experiments utilizing methylene blue (MB) reveal that the composite films exhibit significantly enhanced photoactivity. Notably, the LS-550 sample demonstrates the optimal degradation efficiency of approximately 76% after 60 min of irradiation, markedly outperforming the pure TiO_2_ film (65%). This superior photocatalytic performance is attributed to the optimized anatase crystallinity and favorable phase composition, which provide abundant surface active sites and facilitate the efficient separation of photo-generated electron–hole pairs. The synergistic combination of excellent hydrophilicity and enhanced photocatalytic degradation efficiency endows the composite films with exceptional self-cleaning potential.
- **Interfacial Adhesion and Mechanical Durability:** Nano-scratch testing shows that the La-doped TiO_2_-SiO_2_ composite structure significantly enhances film–substrate adhesion. The improved mechanical performance is attributed to the combined effects of the SiO_2_-containing amorphous phase and the finer microstructure induced by La incorporation, both of which help relieve stress concentrations and enhance resistance to crack initiation and propagation. As a result, the critical load (Lc) of the LS-550 sample reaches 75.64 mN, which is substantially higher than that of the pure TiO_2_ film annealed at the same temperature (61.25 mN), indicating superior interfacial mechanical durability.

In summary, these findings can facilitate the development of high-performance TiO_2_-based coatings with improved structural stability, hydrophilicity, photocatalytic activity, and interfacial durability. Among all the investigated samples, the LS-550 film exhibits the most favorable overall balance of crystallographic thermal stability, interfacial mechanical durability, surface wettability, and photocatalytic degradation performance, indicating promising application potential, particularly in dust-resistant encapsulation coatings for photovoltaic modules [59], self-cleaning glass for high-rise building facades [60], and protective coatings for outdoor optical sensors [61].

## Figures and Tables

**Table 1:** Nomenclature of Experimental Samples.

Labeling of Samples	Composition of the Sample	Annealing Temperature (°C)	Notes
P-550	TiO_2_	550	control group
LS-AD	La, SiO_2_	As-deposited	
LS-350	La, SiO_2_	350	
LS-450	La, SiO_2_	450	
LS-550	La, SiO_2_	550	
LS-650	La, SiO_2_	650	

**Table 2:** Quantitative XRD analysis results of the anatase and rutile phases.

Sample	Anatase Crystallinity Index	Rutile Crystallinity Index
P-550	32.3%	12.4%
LS-AD	—	—
LS-350	28.0%	—
LS-450	45.9%	—
LS-550	49.7%	—
LS-650	41.6%	—

**Table 3:** Roughness Data Sheet.

Sample	Average Surface Roughness (Ra/nm)	Root Mean Square Roughness (RMS/nm)
P-550	1.1989	1.7042
LS-AD	3.2753	4.6231
LS-350	1.3422	1.9187
LS-450	1.0062	1.2508
LS-550	1.0949	1.3683
LS-650	0.9840	1.2194

## Data Availability

The original contributions presented in this study are included in the article. Further inquiries can be directed to the corresponding author.
